# An Industrial Micro-Defect Diagnosis System via Intelligent Segmentation Region

**DOI:** 10.3390/s19112636

**Published:** 2019-06-11

**Authors:** Xia Fang, Wang Jie, Tao Feng

**Affiliations:** School of Manufacturing Science and Engineering, Sichuan University, Chengdu 610065, China; 18215575946@163.com (X.F.); fengtao@scu.edu.cn (F.T.)

**Keywords:** non maxima suppression, intersection over union, simple linear iterative clustering, segmentation quality, predicted masks, parallel K-means, continuous fine-tune

## Abstract

In the field of machine vision defect detection for a micro workpiece, it is very important to make the neural network realize the integrity of the mask in analyte segmentation regions. In the process of the recognition of small workpieces, fatal defects are always contained in borderline areas that are difficult to demarcate. The non-maximum suppression (NMS) of intersection over union (IOU) will lose crucial texture information especially in the clutter and occlusion detection areas. In this paper, simple linear iterative clustering (SLIC) is used to augment the mask as well as calibrate the score of the mask. We propose an SLIC head of object instance segmentation in proposal regions (Mask R-CNN) containing a network block to learn the quality of the predict masks. It is found that parallel K-means in the limited region mechanism in the SLIC head improved the confidence of the mask score, in the context of our workpiece. A continuous fine-tune mechanism was utilized to continuously improve the model robustness in a large-scale production line. We established a detection system, which included an optical fiber locator, telecentric lens system, matrix stereoscopic light, a rotating platform, and a neural network with an SLIC head. The accuracy of defect detection is effectively improved for micro workpieces with clutter and borderline areas.

## 1. Introduction

Along with the advances in virtual-reality and augmented-reality technologies [1,2,3], the user impression of virtual tactile interactions will significantly impact smartphones and smart wearable devices [4,5]. As for the high quality of virtual tactile experiences and long-term working stability of eccentric rotor motors [6,7,8], the quality of the armature is the key factor. Compared with the defect detection of motor armatures of finished products [9], the production line detection based on machine vision is more economical. For the defect detection of non-standard micro workpieces, it is difficult to quantitatively distinguish the defect level and category by classic machine vision technology [10,11,12]. Meanwhile, with the development of visual detection based on artificial neural networks [13,14], in which image features are fed into the region of interest (ROI) for classification and segmentation [15,16,17], the accuracy of detection is constantly improved. The application of multi-scale feature-based fusion improved the capturing ability of detail features in the target image [18,19,20]. Then joint classification and segmentation method [21] and pixel-level tag segmentation improve the performance to the state-of-the-art in image classification [22,23].

The detection objects of armature identified in detection processes include copper wire, soldering tin, and resistance, which are covered by each other. Many fatal defects occur in the clutter, transition and boundary areas, where the image gradient is mixed. Thus, common pixel-level labels cannot effectively mark the defect areas, and the instance/mask level shows low accuracy.

Inappropriate mask estimation will misguide the judgment of a detection neural network. Some unsupervised clustering methods for images with large gradient change and difficult boundary selection have had a good effect on dividing the regions [24,25,26,27,28].

However, it is difficult to achieve a high accuracy in the field of non-standard micro workpiece like armature detection using the previous mentioned methods. The classic machine vision system involves the statistical characteristics in a specific range to classify and distinguish objects, and the determined threshold cannot reach a high robustness for the detection of non-standard workpiece shapes. ROI based network using the anchor point to conduct the regression is also difficult to achieve a high discrimination accuracy due to the large number of irregular defect objects. The most efficient approach is to establish the pixel-level labels for the detected region, so that the neural network can learn the discriminating area and category. However, armature defects often occur at the boundaries of the target region and can directly affect the quality of the eccentric rotor motor.

**Figure 1:**
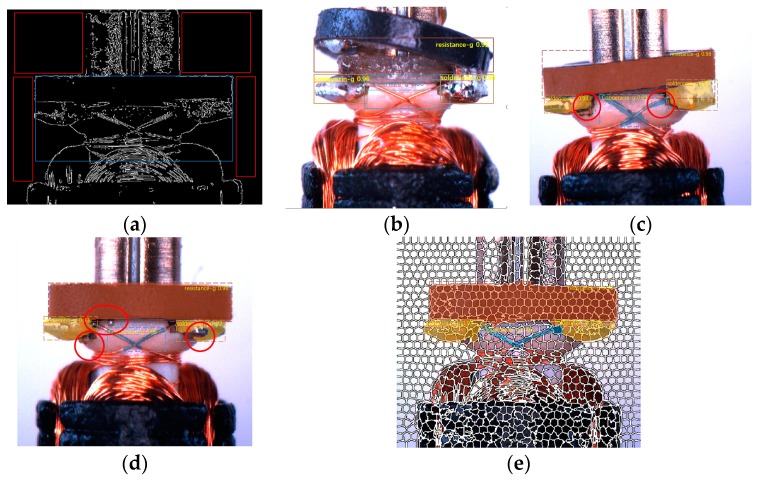
Different methods to detect defection. The traditional method of computer vision, the region-based neural network method of computer vision (upper row), which cannot recognize fatal defects in the red circle, finally comes our method (bottom row). (**a**) The results of the anisotropic diffusion and canny algorithm to obtain the statistical features, (**b**) the results of the Faster R-CNN, (**c**,**d**) shows the results of the object instance segmentation in proposal regions, (**e**) the results of the simple linear iterative clustering (SLIC) head based on Mask R-CNN.

In this work, the simple linear iterative clustering (SLIC) head was proposed to augment the mask area in the fully convolutional networks (FCN) branch [29] of the mask region-based convolutional neural network (R-CNN) [22] and coordinate the predicted score through ROI features [30,31]. The content of the predicted mask is divided into many cell regions with different degrees of overlap, which will complement each other with the ground truth. In order to obtain a better and more efficient fitness of the selected pixels in the mask area, we employed a fixed step size traversal superpixel optimization method [32]. By constantly comparing the attribution of pixels in the cell regions, we augmented the predicted mask. Then we integrated the augmented mask with the features in the ROI to establish regression related to the intersection over union (IOU) [22] value of the augmented mask and ground truth. The mask of the neural network could cover the required detection area more comprehensively. The results of the anisotropic diffusion and canny algorithm to obtain the statistical features by a classic vision method is shown in the red box; however, this method could not be applied to the irregular shapes in the blue box (Figure 1a). The results of the Faster R-CNN [17], in which it is difficult for the discrimination score to reflect the high confidence of the polytropic workpiece are found in Figure 1b. The results of the Mask R-CNN [22], in which the incomplete mask is easy to occur at the boundary and in regions with a large gradient, and the network is not aware of the score misalignment caused by the incompletion are found in Figure 1c,d. The results of the simple linear iterative clustering (SLIC) head that calibrated both masks and scores are found in Figure 1e. 

**Figure 2:**
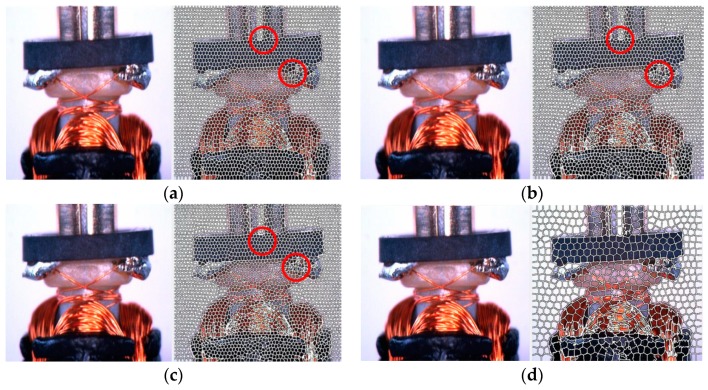
(**a**,**b**) The results of same initial value of the center points with 10 and 15 iterations respectively. (**b**,**c**) The results of same numbers of iterations with different initial values of the center points. (**d**) The result description of bigger size of superpixel cells.

The original armature picture is divided into many superpixel cells by parallel K-means in limited area [18]. The initial center of each iteration in SLIC is randomly distributed at the lowest gradient area of input image, which is illustrated as the distortion of cells in the red circle areas. (Figure 2). The appropriate size of input images is 800 × 1333 pixels, with which superpixel cells *K* = 400 (400 pixels in a superpixe cell) and characteristic viscosity m = 10 (maximum possible distance of the CIELAB (The color space that ICC Profiles and CMMs often use as an intermediary space when converting colors) space as in the Ref. [33]). For the same number of iterations, with SLIC features of different scales after the same proportion contraction, the clustering center deviation was larger at the higher gradient transformation as shown in the red circle in Figure 2. As the mask of the clutter area is frequently missing, mixed and disorderly, the corresponding cell regions are distorted. Figure 2 sketches the misalignment between different superpixel sizes and characteristic viscosities (maximum possible distance of the CIE-lab space [34]). 

The superpixel generated by K-means in the same region will be distorted to different degrees. Different layers use the same number of iterations, resulting in a larger distortion point transformation in a high gradient area, but there is almost no difference in image areas with a small gradient. It can be seen that in the borderline areas and the jumble part of the objects, the gradient changes sharply, so there will be a lot of deviation in the superpixel. We use the superpixel deviation, which has the property of large clustering area; we use this bias to compare the overlap of the SLIC mask with the ground truth to classify pixels that belong to the same superpixel unit into the category of matching objects to augment the mask.

In this work, ROIs were selected with an IOU of more than 0.5 between matched ground truth, and fed into the SLIC head with the predicted mask. Because the gradient changes dramatically in clutter and occlusion areas, the clustering of superpixel elements will show a huge deviation in different layers or in the same feature layer but with different iterations compared with the mask of ground truth. Following the Mask R-CNN [22], the bilinear interpolation samples 2000 recommend areas of the same size and then carries out the IOU [22]. A simple regression method is used to augment the mask between the ROI features with SLIC used to ensure that the network is aware of the quality of the mask. 

Section 2 describes how to use the SLIC head to augment mask selected regions and enhance the robustness of our system for large-scale production lines. Section 3 presents the design of the structure and details the working principles of our system. Section 4 introduces the experimental platform system. Section 5 shows the experimental results. Section 6 provides a conclusion.

## 2. Methodology of the System Implement

In this section, we describe how our SLIC head works for mask augmentation from an armature image. Since this system is used for the world’s second largest motor production line, we need to screen the data for active learning according to the sensitivity characteristics of the network, and undergo continuous fine-tune before learning the inflection point of the learning efficiency curve. 

### 2.1. Mask Augment through SLIC

**Figure 3:**
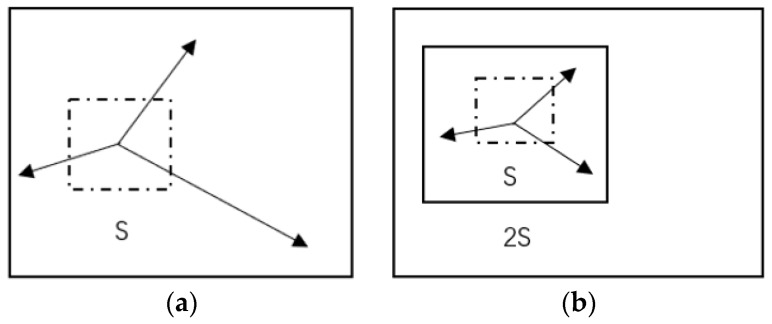
The K-means method is used to generate superpixel cells, while maintaining the relevance of pixel texture, the traversal speed is also guaranteed [36]. Ordinary k-means (**a**) and parallel k-means in the limited areas (**b**).

In the training process of Mask R-CNN, when the matched predicted mask with the highest score of category *K* is output from the FCN branch, a regression value is generated with the matched ground truth. Before that, these predicted masks are fed into the SLIC head to generate SLIC cells of predicted mask and augment them by comparison with the matched ground truth. Different types of masks with SLIC cells are not processed and the method and principle of addition are as follows. Specific masks are fed into the CIE-lab color space to obtain $V_{k}=\left(l_{k},a_{k},b_{k}\right)$ and the space of pixel distance $V_{k}=\left(x_{k},y_{k}\right)$. $l_{k}$ component is used to represent the brightness of the pixel, representing the range from pure black to pure white for the value range of 0 to 100 [35]. $b_{k}$ represents the range from yellow to blue, $a_{k}$ represents the range from red to green, and the value range of both is 127 to 128. $x_{k}$ and $y_{k}$ are the coordinates of the center point $C_{k}$ of the superpixel unit. *K* seed points are generated with the S stride, and then those pixels near by the seed points in the surrounding space of each seed point are selected, classifying them into the same category as the seed point until all the pixel points are classified. The average vector value of *K* pixels in the superpixel is then calculated, so as to regain the *K* clustering center. The surroundings are searched for the most similar number of pixels with the *K* clustering center, after all the pixels are classified to obtain *K* superpixel. The iteration is restarted when the clustering is updated, and repeated until convergence. Figure 3 depicts the *K*-means traversing in the limited area and search the 2S × 2S area, which constitutes a S×S superpixel to improve the speed of each iteration. This approach guarantees the accuracy of each iteration, while also reducing calculation time. Once a pixel has been associated with the nearest cluster center, the updating step adjusts the compact class center to the average vector $V_{i}={\left[l_{i},a_{i},b_{i},x_{i},y_{i}\right]}^{T}$ of all pixels belonging to the cluster. The assignment and update steps can be iteratively repeated until the error converges. We found that 15 iterations were sufficient for most images and we used this standard in this paper. $L_{i}$ is the label of the superpixel and S is the stride size used for generating superpixel cells.

(1) $$S=\sqrt{\frac{N}{h}}$$

where N is the number of pixels contained in the segmented graph and *h* is the number of superpixel units.

In the process of integration, comprehensive pixel size and characteristic viscosity state distance are used to describe the matching relationship between vectors. The clustering center moves to the location of the minimum gradient in *3S × 3S*, corresponding to each pixel set label $L_{i}=-1$, and each pixel set distance $d_{i}=\infty$. The *D* norm is used to calculate the residual *E* between the new cluster center and the previous cluster center. Calculating the distance of each one pixel between $C_{k}$, when *D* <$\;d_{i}$, the set $L_{i}=k$, $d_{i}=D$ to update pixels into the cluster. Then calculate the residual *E*; stop iteration when *E* is less than the threshold. We classified the area with the same tag of pixels as a super pixel block, denoted as $C_{1},C_{2},C_{3},\ldots C_{k}$, where *K* is the number of super pixel blocks. The number of super pixel blocks is decided by seed number, image size, and image color complexity. The number of seed points was set to 400 by default. 

**Figure 4:**
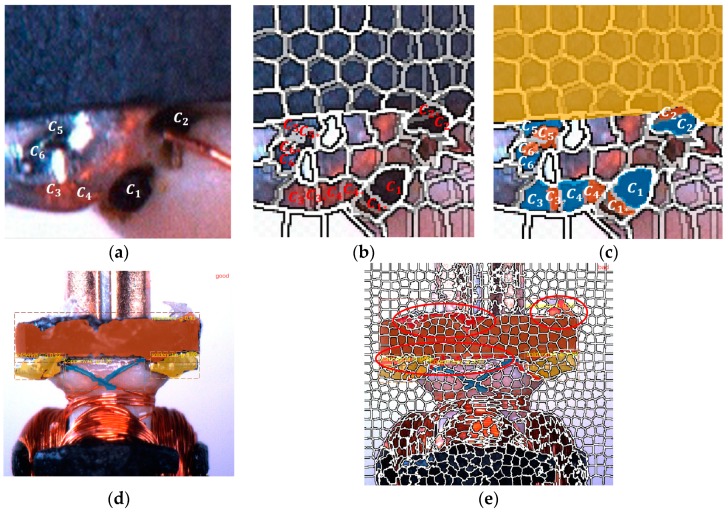
$C_{1},C_{2},C_{3}\ldots C_{6}$ are the clutter detection area (**a**–**c**). The trained model to detect and segment three objects show in the (**d**,**e**), which as shown in the red circle, the red labeled pixels are reincorporated into the labeled simple linear iterative clustering (SLIC) cells to achieve augmentation of the mask. (**a**) Shows the original image with defects, (**b**) describesthe deviation caused by superpixel segmentation, (**c**) shows the process of augmentation, (**d**) illustrates the segmentation method based on mask, (**e**) shows the result of our method.

In order to avoid the unreasonable pixel points such as edge and noise and the selected clustering center, the algorithm was modified. In the windows of *3S × 3S,* the clustering center was moved to the region with the minimum gradient, and the gradient ($g_{i}$) is fitted with a function of the form

(2) $$g_{i}(x_{i},y_{i})={\left[V_{i}(x_{i+1},y_{i})-V_{i}(x_{i-1},y_{i})\right]}^{2}+{\left[V_{i}(x_{i},y_{i+1})-V_{i}(x_{i},y_{i-1})\right]}^{2}$$

because $V_{k}=\left(l_{k},a_{k},b_{k}\right)$ is limited in the CIE-lab color space, color size is limited, while the image size is not. If the image size is large, the space distance $V_{k}=\left(x_{k},y_{k}\right)$ will have too much influence in the measurement of vector distance, thus the space distance $\left(x_{k},y_{k}\right)$ needs to be modulated. To normalize $\left(x_{k},y_{k}\right)$, the improved vector distance is measured as follows

(3) $$d_{c}=\sqrt{{\left(l_{j}-l_{i}\right)}^{2}+{\left(a_{j}-a_{i}\right)}^{2}+{\left(b_{j}-b_{i}\right)}^{2}}$$

(4) $$d_{s}=\sqrt{{\left(x_{j}-x_{i}\right)}^{2}+{\left(y_{j}-y_{i}\right)}^{2}}$$

(5) $$D=\sqrt{{d_{c}}^{2}+{\left(\frac{d_{s}}{S}\right)}^{2}m^{2}}$$

where $d_{s}$ represents the pixels in the *5D* coordinate mapping space distance, $d_{c}$ represents the color of the pixel in the *5D* coordinate mapping proximity, m is the maximum possible distance of the standard color space (LAB space) and s for the largest possible distance of the *XY* space. The post-processing step implements connectivity by assigning a new non-intersecting pixel face to a nearby superpixel. Compared with the matched ground truth, every pixel in the cell of superpixel induced to the nearby seed points of cells through the difference values variable of different iteration, then the area is augmented. By comparing the pixels in SLIC cells of predicted mask with the ground truth, it was found that if the overlap gap of a corresponding superpixel unit distinction is more than 30% on average (Figure 4), the pixels will be included in the classification.

By using this mechanism, the mask of detection areas is augmented and the final result of discriminant is corrected.

In this work, the dataset was pixel-level labeled. Three label types are copper wire, soldering tin, and resistance. Copper wire-g (good), pixel label is present by blue color; copper wire-d (defection), pixel label is present by blue color; soldering tin-g (good), pixel label is present by yellow color; soldering tin-d (defection), pixel label is present by yellow color; resistance-g (good), pixel label is present by orange color; resistance-d (defection), pixel label is present by orange color. The output value is a binary classification between good and bad. If any one of these defects was predicted, we determined whether the workpiece was actually defective.

When the pixel category in the same cell is 30% different from the pixel category in the matched predicted SLIC mask of the same category center $C_{i}$, $C_{i}$ is classified into the category closest to the center. A complementary SLIC mask is finally formed after comparisons.

We pre-processed the dataset with the corresponding images and input them into a pre-trained neural network (ResNet101-FPN [37] on COCO (A standard illegible dataset.) [38]). A predetermined ROI was set for each point in the feature map to obtain multiple candidate ROIs. Then, these candidate ROIs were sent to the regional proposal area (RPN) network for binary classification (foreground or background) and bounding box regression to filter out some of the candidate ROIs. Next, we selected the proposed ROIs by IOU. If the IOU value was more than *0.5*, the ROI SLIC operation was performed on the remaining ROIs, which were fed into the SLIC head. We subsequently used softNMS [39] to select ROIs for the mask head; processing details are described in the next section. Here we used the distance relationship of the LAB (A color division space based on chroma and brightness as units.) coordinate system, the hex-code color initial center, and the continuity detection methods [40]. 

In the process of superpixel augment, the positions with drastic changes in color texture and spatial distance will be supplemented, and the superpixel deviation is not large in the flat gradient regions. We selected images with an IOU of more than *0.5* with ground truth for the SLIC head training, and conducted pixel level regression of the same category. The generated superpixels are compared with the superpixel of ground truth. If the distances of the same or adjacent superpixel cells are different, the superpixel in the region will be planned as this category. Through repeatedly screening and comparison, the final classified super pixel mask and super pixel label are used to obtain the loss value

### 2.2. Data Processing Process of the System 

In the dataset we established, the defect components were miscellaneous. The detection characteristics are difficult to be defined and cannot be accurately labeled when making dataset, which exist in most non-standard workpiece computer vision detection systems. The application object of our system is the world’s second largest micro motor production line, so the robustness of the system will face a significant challenge. However, according to the elbow method [41], there is no strict positive correlation between the cognitive ability of the system and the size of the dataset. Meanwhile, because of the high cost of tagging dataset in a Mask R-CNN type network, we needed to find the most sensitive objects of the network in the limited dataset and draw clear boundaries between defect classes. We used the Mask R-CNN model pre-trained on COCO dataset [38] and used continuous fine-tune [42] of the whole model with a ResNet-101 backbone. 

**Figure 5:**
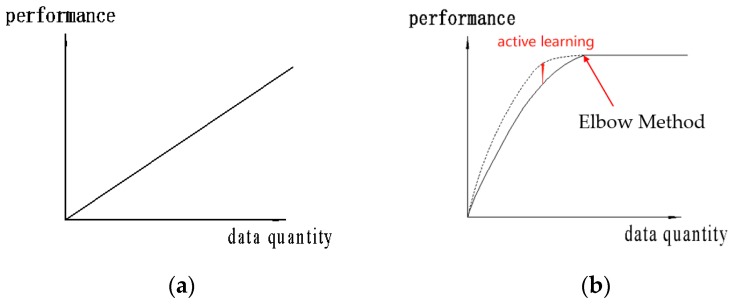
The performance of the model based on statistical characteristics increases linearly with the increase of data (**a**). When the data volume reaches a certain quantity the performance of the deep learning model, of which data sensitivity will reach a bottleneck and will not obviously increase with the increase of training data (**b**).

Here, we proposed the use of active learning to fine-tune the model. Figure 5 shows the notion of “bigger is better” is not conductive to the nonlinear model dataset, and the learning performance has certain limitations with the enhancement of dataset.

**Figure 6:**
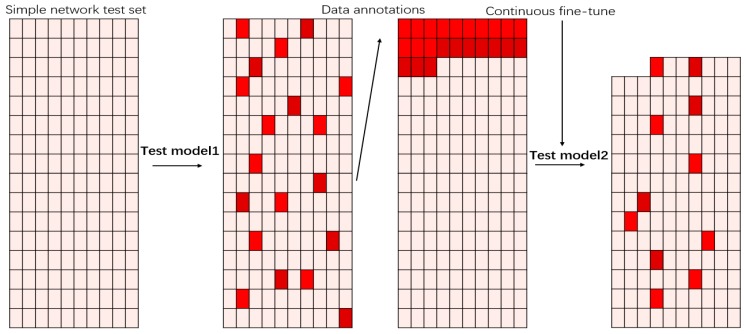
Red data indicates that the discrimination probability of the neural network is around 0.6. We extracted these datapoints and labeled them as a new dataset to the fine-tune neural network.

In the case of a large amount of object data to be processed, active learning achieves a high robustness by using as little as possible annotated data. First, we trained a starting model with a high level of confidence defect artifact dataset, in which it is easy to distinguish good from bad. Second, we continued to input the unlabeled test set into the model. We marked the data with score values between *0.6* and *0.65* and fed them into the dataset for the fine-tune process of the model. According to the probability values in the network, we finally identified what kind of defect data model network was more difficult to be distinguished. Figure 6 presents the continuous updating of the model, our network gained a higher robustness under the limited dataset.

Moreover, for future detection in a production line, data with probability values between *0.6* to *0.65* should be input into the dataset to be annotated next time, so as to continuously increase the accuracy of the model. The fluctuation of our dataset will be huge in terms of network classification and recognition probability; thus, the values in the vicinity of *0.6* in softmax are considered as high entropy of the data. We screen out such data, and enhance them by image flipping, stretching, and adding gaussian noise to image. By operating a few repetitions, constituting a blend of workpiece training dataset, we can produce the final discriminant network. 

In the process of detection in the production line, extraction workpieces with discriminant values between *0.6* to *0.65* were input into the dataset for active learning. Eliminating the workpieces with ambiguous defects, fed the workpieces into training sets, which can be easily distinguished from the defect types of corresponding labels. After reselected, every 30,000 workpieces updated the parameters of the model, and executed continuous fine-tune. When the training sets expansion up to 120,000, the elbow method [30] happened, so we extracted the model as the final version of the network. Then 30,000 reselected workpieces combined with an original 90,000 workpiece into 120,000 new dataset, among which the trained dataset accounted for *70%* and the test dataset for *30%*. 

## 3. Implementation Details of SLIC Head

In this work, the backbone was the ResNet101-FPN (The 101 layers residual neural network is used in combination with the feature pyramid network.) network [28], corresponding to the characteristics of the pyramid, which improved the detection accuracy to a certain extent. The FPN network in our work included conv4_x, and 23 blocks of 14 × 14, with catch features efficiently matched with SLIC cells. 

**Figure 7:**
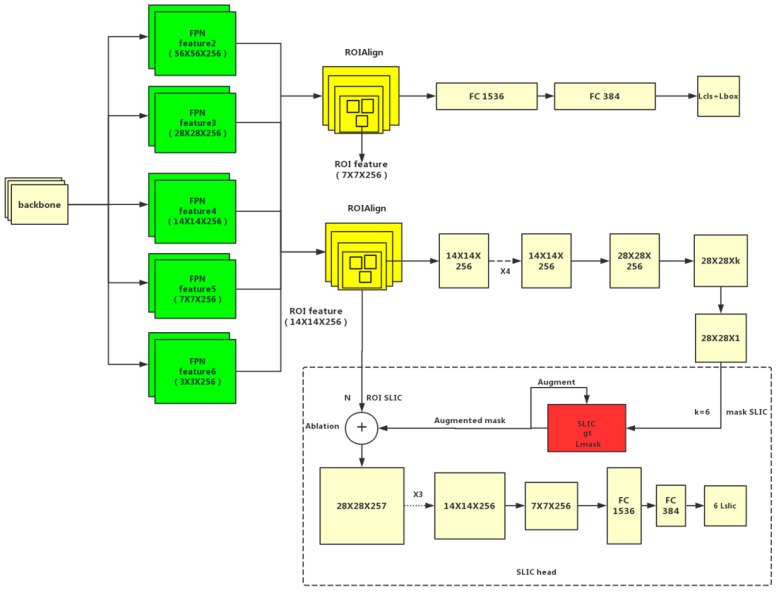
Structure diagram of the system is generated for the SLIC head.

Due to the previous multiple convolution and pooling, the corresponding resolution was reduced. Following Mask R-CNN, ROI align [16] removed the harsh quantization of the ROI pooling, aligning the extracted features with the input properly, which avoid quantization missing of the ROI boundaries or bins. Bilinear interpolation was used to compute the exact values of the input features at four regularly sampled locations in each ROI bin, and the results were aggregated. Noting that the results are not sensitive to the exact sampling locations or how many points are sampled, as long as no quantization is performed. The mask branch used deconvolution to improve the resolution and reduce the number of channels to 28 × 28 × 256, while the R-CNN branch had dimensions of 7 × 7 × 256. Finally, the mask template of 28 × 28 × C was output. The ROI region features in different RPN were mapped to the region of 14 × 14 through bilinear interpolation, which was compared with the original features in input images to generate accurate superpixel cells. Figure 7 shows the SLIC head has three convolution layers of 28 × 28 × 257 (it’s in the dotted box at the bottom of the Figure 7), two convolution layers structure of 14 × 14 × 256, 7 × 7 ×256 and three FC layers (Dimensions are 1536,384 and the final FC outputs six classes that generate superpixel regression loss function between the ground truth and augmented mask). Left and right soldering tin are in one category because we mirrored the image to the expanded dataset.

Features of various scales are carried out by ROI alignment. Then we used a simple cascading structure to input the augmented mask and ROI SLIC features into the SLIC head as shown in Ref. [43]. The IOU of the input ROI features between matched ground truths for the SLIC head are more than *0.5*, which is the same as the training sample of the Mask head of the Ref. [22]. Superpixel regions are generated in the feature graph of up-sampling to the SLIC-features from ROI original features in input images. 

SLIC head learned the regression value between the augmented mask and the matched ground truth through ROIs SLIC and the augmented mask. 

The Mask R-CNN has four loss terms in equal weights—a pair of loss function for RPN; a softmax detection loss for C + 1 categories classing; a softmax segmentation loss over the foreground mask of the ground-truth category only; and a bbox regression loss. The latter two loss terms are effective only on the positive ROIs. $L_{cls}$ and $L_{bbox}$ are the same as those in the Mask R-CNN. For each ROI, the mask branch has N∗k dimension outputs, and the calculation method of $S_{cls}$ in $L_{mask}$ is unchanged. Meanwhile encodes N cell regions with a size of 400, and each cell region has k categories. We used per-superpixel sigmoid and defined the $L_{slic}$ as the average binary cross-entropy loss. $L_{slic}$ is defined only on the *k* cell regions (other *k-1* mask outputs do not contribute to the entire loss, and soft non-maximal suppression was used). Then we connected the mask head branch to the SLIC head and expressed it as a simple loss relation function:

(6) $$L=L_{RPN}+L_{cls}+L_{box}+L_{mask}+L_{slic}$$

The entire system structure of this paper is shown in Figure 7. 

We used the $L_{2}$ loss for the regression between the SLIC mask and the ground truth, and the loss weight was set to 1. The proposed SLIC head was integrated into the Mask R-CNN, and the whole network was trained end to end. The method of ablation is according to Ref. [43]. During the inference of our system, an augmented mask score $S_{slic}$ was generated in the SLIC head based on the predicted SLIC mask and ROI features.

Therefore, after calculating the regression loss value, we only needed to calibrated the original $S_{mask}$ with the $S_{slic}$ to ensure that the network was aware of the quality of the augment mask after the SLIC head [44].

For example, we output the score value of the copper wire augmented mask by SLIC head, as shown in Formula (7).

(7) $$S_{slic-mask-copperwire}=S_{mask-copperwire}S_{slic-copperwire}$$

This way, in the process of rotor defect detection of micro vibration motors, the neural network can be aware of whether the appropriate detection region is selected, especially when the workpiece has a complex transition region and an uneven diffuse reflection region.

We used the scores of the SLIC head to adjust the classification score generated from the mask branch for inference. Specifically, suppose the R-CNN stage of the Mask R-CNN outputs 512 bounding boxes, and among them the top 200 scoring boxes after non-maximum suppression algorithm with weight distribution (SoftNMS) [39] are selected. Then, the top 200 boxes are fed into the SLIC head with a generated fusion ROIs SLIC feature and fed into the matched target augmented masks to predict SLIC mask confidence. The branches of the mask were all based on the 200 predicted results with the highest score, so the added calculation amount was small.

The images of those IOU between predicted masks and ground truth more than 0.6 were selected to pre-train the SLIC branch, then added to the branch of network for overall training.

## 4. System Apparatus and Related Work

We built a complete system for real-time shooting, workpiece positioning, active learning, model training, and model application for the detection of rotating micro-workpieces. Because the workpiece under inspection was a three-phase two-brush direct current motor armature, it needed to be positioned at multiple angles. This system was aimed at the rotor part of the eccentric vibration motor. 

**Figure 8:**
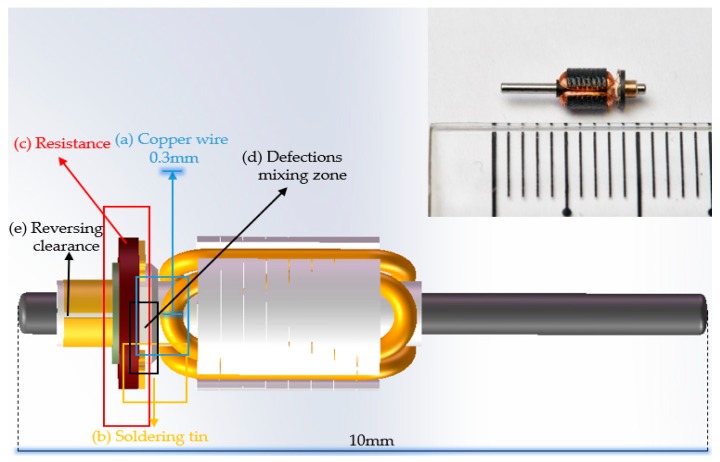
Present the main targets of our detection (**a**–**c**), the region prone to many miscellaneous fatal traps (**d**), (**e**) the commutation gap.

The position of reversing clearance of the commutator at each detection surface of armatures was captured by fiber optic sensor (E32DC200B4, OMRON, Kyoto, Japan) and fiber optic amplifier (FX-101, OMRON, Kyoto, Japan). There were three main defects in the rotor, each of which would lead to serious faults in the use of the motors. We only marked one side of soldering tin due to flipping the images to increase the numbers of dataset. Crack, foreign matter, leveling of resistance, welding of copper wire, and cold joints of soldering tin were seriously affecting the working state of the motor. Impurities between various regions would directly lead to short circuiting of the motor and even damage the circuit of the devices; the defects at theses border regions were the most fatal to the motor. The remaining defects were identified by the region of captured characteristics based on statistics features, which were independent of our network. The defect detection target of this work is shown in Figure 8.

**Figure 9:**
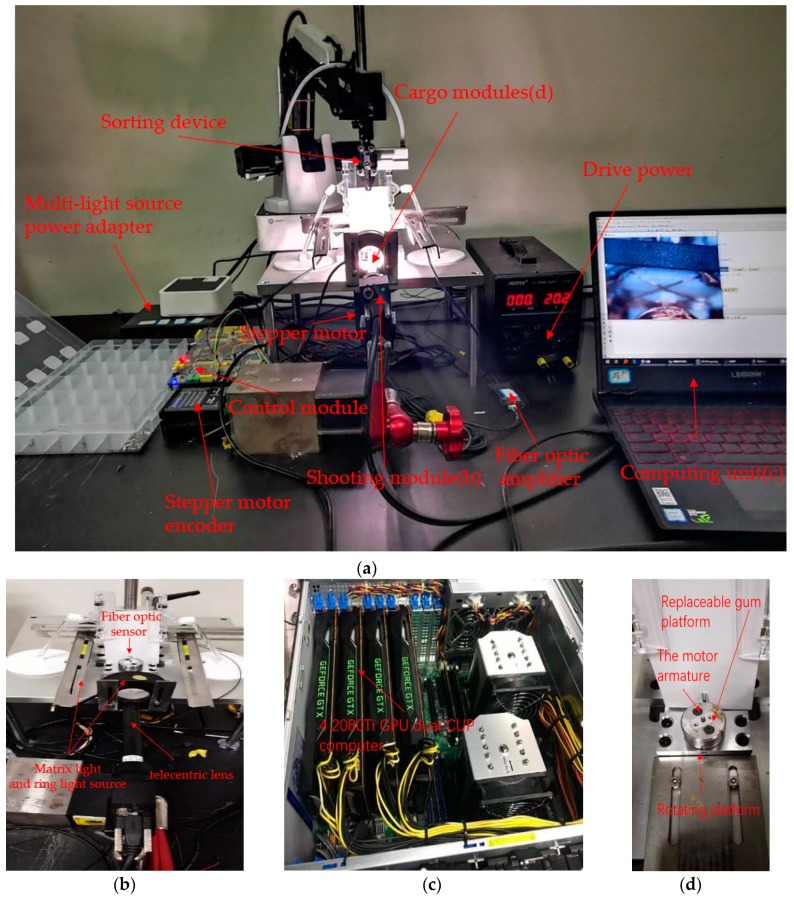
(**a**) The control module, sorting device and the light source group. (**b**) The matrix light and ring light source, as well as the laser source to provide a feedback signal for the stepping motor. (**c**) Two computers—one for net training and another store model for portable use in the production line. (**d**) The rotating platform.

A proper illumination system was used to ensure the high quality of the image, which included two matrix light sources, and a ring light source. The ring light source was arranged in front, two matrix light sources were arranged at the left and right. We used the plane-array 2/3 CMOS camera (Basler, Munich, Germany) with 1.3 million pixels and the telecentric lens with 0.66 mm depth of field, 110 mm working distance to ensure the magnification and ensure the field of view is large enough. Thus, our system was not disturbed by the external light source. In order to ensure the universal property of the system, replaceable polyacetal, polyoxy methylene (POM) fixture, and the replaceable platform of the armature were used to readjust the posture of the workpiece. After the discrimination, the workpiece was placed by the sorting device. Figure 9 shows the details of the system.

We trained the whole network for 24 epochs and the base learning rate was 0.001. The learning rate decays by 10% at turns of 16 epochs and 20 epochs. We used the saccharomyces genome database (SGD) as the optimizer and the momentum was 0.9. During the accuracy testing phase of the model, we used SoftNMS [39] to retain the top-100 score of ROIs for each image, extracted the highest score in the same category as output. The ResNet-101 configuration followed the detector Ref [16]. The conditions of our experiment included hyperparameters (using the existing MASK-R-CNN, SLIC head), a backbone structure (ResNet101-FPN),an input image (resized to 800 pixel for the short axis and 1333 pixel for the long axis), and four 2080TiGPU@1 images for training with mini-batch equal to 4.

The software system was programmed in Python. The detection algorithm was developed by OpenCV and Tensorflow deep learning platform, imds were produced by matlab Label tool.

## 5. Results

We validated our module on our own dataset and achieved the final detection accuracy of 98.8%. Defective parts on the armature were detected when at least one label detected a defection. Because of the specificity of our subject’s defect type discrimination, the lack of a mask in the foreground area can seriously affect the accuracy of the defect detection. Our comparison groups selected R-CNN class models that performed well on other datasets, as well as the statistical features discrimination for the defined area. 

As our content involved detection of good and defection samples, the accuracy of this model is constituted by true positive (TG), true negative (TD), false positive (FG), and false negative (FD) value [44] by

(8) $$Accuracy=\frac{\left(TG+TD\right)}{\left(TG+TD\right)+\left(FG+FD\right)}$$

**Figure 10:**
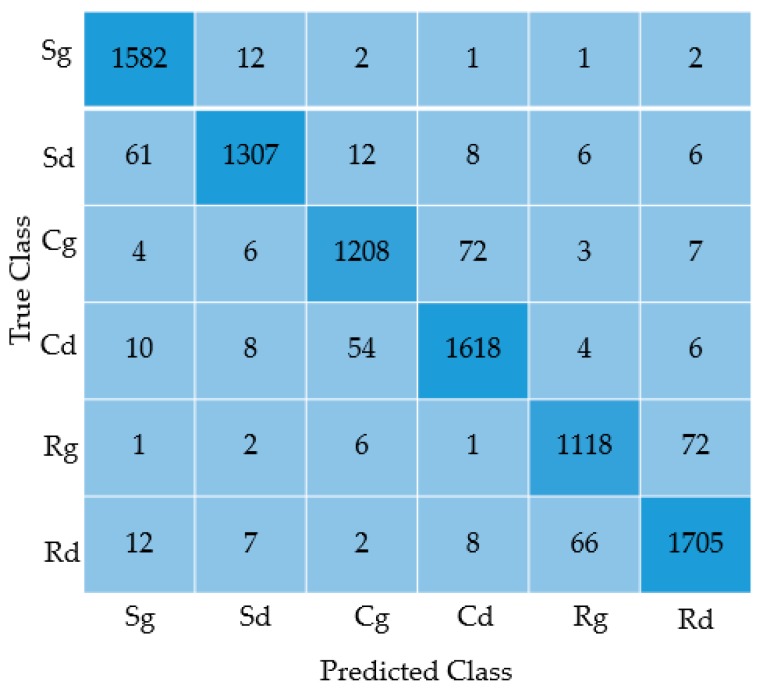
The confusion matrix for results of the 9000 test dataset for three types of defections.

Several comparisons were made according to different conditions in this paper, which proved that the parameters selected in this paper were feasible, shown in Table 1. Figure 10 shows the misclassified case, which is represented by the confusion matrix of the 9000 test dataset in the first 30,000 dataset. The quantity of defective samples of three types of workpieces is different from that of good samples, the number of different categories are: Soldering tin (1600 good and 1400 defect), copper wire (1300 good and 1700 defect), resistance (1200 good and 1800 defect). 

**Figure 11:**
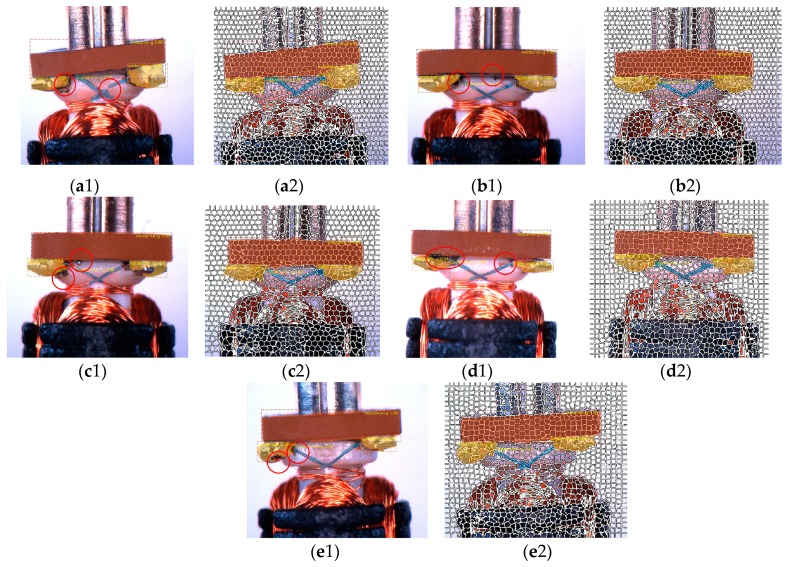
The augmentation effect of the mask in detected areas. (**a1**–**e1**) Examples of inaccurate defect detection due to incomplete mask, (**a2**–**e2**) compare the results of our method for the same artifact.

Table 2 displays the comparison between the results from mask R-CNN to results after being processed by our system. In this paper, we picked out about 30,000 sensitive samples from the production line every month, to enhance the existing dataset. Using the continuous fine-tune method, the accuracy rate on the production line after two months reached 98.8%, following repeated tests by employees. It can be seen that some areas with large gradient changes are prone to missing the mask, and these missing parts contain very important detection information. The quality problem for the mask itself causes the confidence level of the model regional recognition score to be low (Figure 11a). Being calibrated by our system the mask gets a better coherence. From the red circles in Figure 10 we can see that in the defect areas after SLIC head completion, the workpiece criterion was more exactly (Figure 11b–e). 

**Figure 12:**
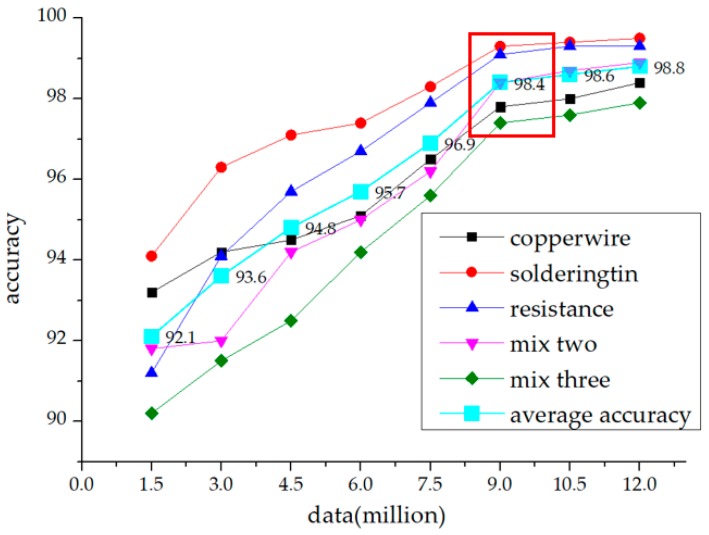
The elbow method happened when the samples were collected to 90,000.

Every 30 days the 30,000 clear data can be classified from the production line, which were sensitive to the model for continual training. The elbow method appeared in 9–12 million of data, so we stopped reselect after 12 million of data. Because of the initial training set, data itself is difficult to calibrate and divide and not clear, thus the accuracy was not high, but after several times of active learning, the network achieved a good result for this kind of motor armature. In the end, we used the latest batch of 120,000 dataset after 30,000 data were added. The result of the final training, and the accuracy, reached 98.8% (Figure 12).

## 6. Conclusions

In this work, the SLIC head is proposed to augment the predicted mask and calibrate score of the mask. The proposed method effectively increased the integrity of the mask and the detection accuracy of three different defects for armatures, exhibiting strong robustness in large scale production lines. As for the original dataset, the accuracy of our model reached 93.6% and the location of defect areas and defect types could be marked out precisely. In addition, after continuous data screening in the production line, the accuracy of the model (through continuous fine-tune) reached 98.8% when the elbow method happened. We built a stable machine vision inspection system that can continuously increase the accuracy in production lines, and achieved good results in actual production. This method has high robustness in the detection of micro-workpiece defects, ensures good detection speed, and can achieve good results in practical engineering applications. A series of experiments with our SLIC head was conducted, and the results demonstrate that our method provides consistent and noticeable performance improvement attributed to the alignment between the mask quality and score. In the field of non-standard workpiece vison defect detection, a better application was achieved. To make this system more universal in the field of non-standard workpiece detected by computer vision, we should adjust the size of the superpixel cell to be more comprehensive. In summary, the main contributions to this work are highlighted as follows:

1. An armature defect detection system for eccentric rotor motors was built. The replaceable fixture in the system was made of POM, which made the system more universal. In the field of non-standard workpiece vision detection, a better application will be achieved.
2. The method of mask augmentation based on the superpixel element decomposition contour was proposed to improve the accuracy of selecting the mixed region with high gradient. The mask confidence is also adjusted for this class of workpiece.
3. We build a dataset processing system that improved the system robustness with the increase of the detection numbers.

Due to some data being prone to controversy, it is hard for us to label them correctly. It is necessary to find a comprehensive process to quantitate the label marking problem. Then in the future, we will improve the model accuracy and the ability of active learning and apply the system in more fields. 

## 7. Patents

A visual detector for defect detection of Eccentric vibrating motor, A notice of acceptance has been obtained.

## Figures and Tables

**Table 1:** We compared the accuracy of models with different sizes of superpixel cell (**a**) and with different overlap areas of superpixel cell (**b**).

(**a**)	
**Size of Cell**	**Accuracy (%)**
300	91.7
350	93.1
400	93.6
450	92.5
500	92.6
(**b**)	
**Overlap Area (%)**	**Accuracy (%)**
20	92.7
30	93.6
40	91.6
50	88.5

**Table 2:** The characteristics of traditional machine vision capture detection methods are not defined by the standard parts, especially in many tests to ensure robustness. In the R-CNN class network, due to the particularity of the non-standard parts, characteristics of the mask in absent places is important, so our network achieved a better effect, as shown by the constant expansion of the dataset, with the help of further expansion effect.

Defect Type	SLIC Head + Mask R-CNN	SLIC Head +Mask R-CNN Expansion 30,000 Data	Statistical Features	Faster R-CNN	Mask R-CNN
Accuracy (%)					
Copper wire	94.2	95.1	75.6	90.2	94.1
Soldering tin	96.3	97.4	82	92.3	92.4
Resistance	94.1	96.7	85.1	93.1	91.4
Mix two	92	95	68.5	88.5	92
Mix three	91.5	94.2	61.5	90.2	91
Average accuracy (%)	93.6	**95.7**	**74.5**	**90.9**	**92.2**
